# PKR: A Kinase to Remember

**DOI:** 10.3389/fnmol.2018.00480

**Published:** 2019-01-09

**Authors:** Shunit Gal-Ben-Ari, Iliana Barrera, Marcelo Ehrlich, Kobi Rosenblum

**Affiliations:** ^1^Laboratory of Molecular and Cellular Mechanisms Underlying Learning and Memory, Sagol Department of Neurobiology, University of Haifa, Haifa, Israel; ^2^Laboratory of Intracellular Trafficking and Signaling, School of Molecular Cell Biology & Biotechnology, The George S. Wise Faculty of Life Sciences, Tel Aviv University, Tel Aviv, Israel; ^3^Center for Gene Manipulation in the Brain, University of Haifa, Haifa, Israel

**Keywords:** PKR, protein synthesis, learning and memory, signal transduction, metabolic stress, aging, cancer, Alzheimer’s disease

## Abstract

Aging is a major risk factor for many diseases including metabolic syndrome, cancer, inflammation, and neurodegeneration. Identifying mechanistic common denominators underlying the impact of aging is essential for our fundamental understanding of age-related diseases and the possibility to propose new ways to fight them. One can define aging biochemically as prolonged metabolic stress, the innate cellular and molecular programs responding to it, and the new stable or unstable state of equilibrium between the two. A candidate to play a role in the process is protein kinase R (PKR), first identified as a cellular protector against viral infection and today known as a major regulator of central cellular processes including mRNA translation, transcriptional control, regulation of apoptosis, and cell proliferation. Prolonged imbalance in PKR activation is both affected by biochemical and metabolic parameters and affects them in turn to create a feedforward loop. Here, we portray the central role of PKR in transferring metabolic information and regulating cellular function with a focus on cancer, inflammation, and brain function. Later, we integrate information from open data sources and discuss current knowledge and gaps in the literature about the signaling cascades upstream and downstream of PKR in different cell types and function. Finally, we summarize current major points and biological means to manipulate PKR expression and/or activation and propose PKR as a therapeutic target to shift age/metabolic-dependent undesired steady states.

## Introduction

**FIGURE 1:**
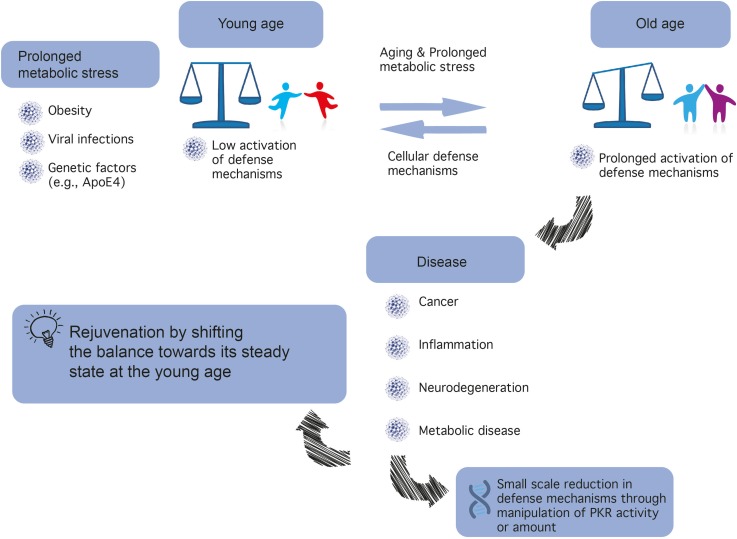
A different balance in defense mechanisms exists in different cells. The development of novel PKR inhibitors differing in properties (e.g., affinity, reversibility) may be advantageous for the treatment of different types of cancer, brain diseases, inflammatory, and metabolic diseases.

Protein kinase R (PKR) is a serine-threonine kinase (551 amino acid long) encoded in humans by the EIF2AK2 gene [located on chromosome 2 (Feng et al., 1992)], which plays a major role in central cellular processes such as mRNA translation, transcriptional control, regulation of apoptosis, and proliferation (García et al., 2007). In accordance with such preponderant role, PKR dysregulation (see Figure 1) has been implicated in cancer, neurodegeneration (Segev et al., 2013, 2015; Stern et al., 2013), inflammation, and metabolic disorders (Segev et al., 2016; Garcia-Ortega et al., 2017). This kinase, which is constitutively and ubiquitously expressed in vertebrate cells, is not found in plants, fungi, protists, or invertebrates (Taniuchi et al., 2016). PKR was first cloned in 1990 at the Pasteur Institute (Meurs et al., 1990; Watanabe et al., 2018), and is also known as Protein kinase RNA-activated; and interferon-induced, double-stranded RNA-domain kinase (Hugon et al., 2009).

The structural composition of PKR consists of an N-terminal double stranded RNA binding domain composed of two tandem repeats of a conserved double stranded RNA binding motif (dsRBM1 and dsRBM2) interspaced by a 23 amino acid linker, and followed by a flexible linker connecting to a C-terminal kinase domain (Meurs et al., 1990). Both dsRBMs are required for the high-affinity interaction with double stranded RNA (dsRNA) (McKenna et al., 2006). The catalytic domain of PKR, where its dimerization takes place, has a typical protein kinase fold formed between its β-sheet N-terminal lobe and its α-helical C-terminal lobe (Dzananovic et al., 2018). However, while the catalytic domain structure is similar to other protein kinases, the interaction of PKR with its best-characterized substrate, the eukaryotic initiation factor 2α (eIF2α), requires a specific α-helix unique to PKR (αG), which is located on the surface of the C-terminal lobe of the kinase domain (Dar et al., 2005).

While the best-described transcriptional motif in the PKR promoter is an IFN-stimulated response element (ISRE), allowing it to be transcribed in response to type I IFN (Kuhen and Samuel, 1997), numerous transcription factors have been identified as binders of the promoter region of the EIF2AK2 gene [e.g., 92 different factors identified by CHIP-Seq assays in the context of the ENCODE project (Rouillard et al., 2016)]. This scenario supports the notion of PKR as an interferon stimulated gene (ISG), while also allowing for the modulation of PKR expression in cellular programs involving the activation of different repertoires of transcription. Activation of PKR results in a number of conformational changes, the most important of which is its homodimerization, based on biochemical and genetic analyses (Dey et al., 2005). As a result of its homodimerization, PKR is autophosphorylated at multiple serine and threonine sites, including Ser242, Thr255, Thr258, Ser83, Thr88, Thr89, Thr90, Thr446, and Thr451 (Taylor et al., 2001). The latter two, namely, the Thr 446 and Thr 451 sites, are consistently phosphorylated during PKR activation, resulting in further stabilization of its homodimerization and increased catalytic activity (Hugon et al., 2009; Watanabe et al., 2018).

**FIGURE 2:**
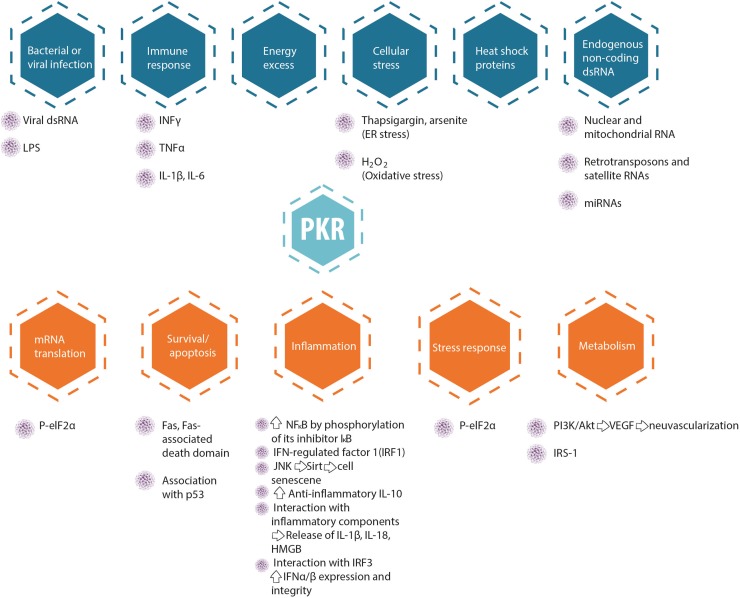
Upstream regulators and downstream targets of PKR.

**FIGURE 3:**
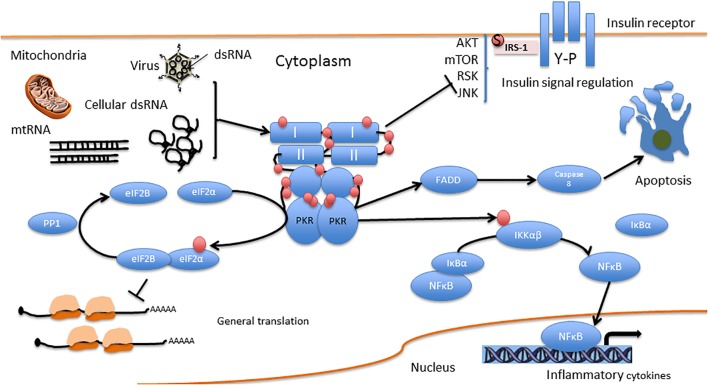
PKR direct interactions. I and II are the dsRNA binding domains. Red circles represent phosphorylation residues.

Protein kinase R serves as a central hub for the detection of cellular stress signals and response to them, and is thus expected to be regulated by different stress-response pathways. In accord with this notion, the canonical activator of PKR is double-stranded RNA (an obligatory feature of the replication process of RNA viruses), rendering PKR as a pattern recognition receptor endowed with cell function modulatory abilities. The central role of PKR in mediating anti-viral responses is also evidenced by the high degree of positive selection exhibited by coding sequence, indicative of the arms race against the pathogens it encounters and combats (Elde et al., 2009; Rothenburg et al., 2009; Carpentier et al., 2016). However, PKR can also be activated by other factors, for example, heat shock proteins, growth factors (e.g., PDGF), and heparin (Li et al., 2006). PKR is also activated in response to numerous insults, including non-viral pathogens (bacterial lipopolysaccharide, which activates the toll-like receptor 4 pathway), nutrition or energy excess, cytokines (e.g., TNF-α, IL-1, IFN-γ), calcium, reactive oxygen species, irradiation (presumably by inducing DNA damage), mechanical stress, and endoplasmic reticulum stress resulting from the presence of a large quantity of unfolded proteins [caused, e.g., by tunicamycin, arsenite, thapsigargin, or H_2_O_2_, which in turn activate the PKR activator protein (PACT; RAX in mice)] (Gil and Esteban, 2000; García et al., 2007; Hugon et al., 2017; Watanabe et al., 2018). Figure 2 summarizes molecular pathways upstream and downstream of PKR, and Figure 3 presents interaction partners and substrates of PKR.

PKR is one of four kinases that regulate protein synthesis via the eIF2α pathway. These kinases include, apart from PKR, the (PKR)-like endoplasmic reticulum kinase (PERK); general control non-derepressible 2 kinase (GCN2), and heme-regulated eIF2α kinase (HRI). All four kinases regulate the phosphorylation of eukaryotic initiation factor 2 on its α subunit (eIF2α), a major regulator of the initiation phase of mRNA translation, the rate limiting step of protein synthesis. Phosphorylation of eIF2α on Ser 51 by any of the four kinases leads to its inhibition and a consequent transient suppression of general protein synthesis, up to its complete blockade, concomitant with translation of mRNAs that encode for antiviral factors and/or mediate the integrated stress response (Hoang et al., 2018). Such blockade of protein synthesis results in the decrease or prevention of viral replication, and may result in apoptosis (García et al., 2007). PKR can also induce apoptosis independently of eIF2α phosphorylation, by activation of the FADD/caspase-8/caspase-3 and caspase-9 APAF pathways (Gil et al., 2002; von Roretz and Gallouzi, 2010).

Both PKR-dependent apoptosis strategies, either with or without blockade of protein synthesis, serve as anti-viral responses. Consequently, many viruses have developed mechanisms which prevent the establishment of an anti-viral state, by inhibiting components of the PKR pathway. These mechanisms include viral proteins that serve as inhibitors of PKR, which inhibit it by direct binding of PKR (thereby preventing autophosphorylation; e.g., Hepatitis C virus, Herpes simplex 1, and Kaposi’s sarcoma vIRF-2), changing its subcellular localization (e.g., Human and Murine Cytomegalovirus), directing it for degradation (e.g., Rift valley fever virus), or regulating its activity. Regulation of PKR activity is done by expression of proteins that disrupt PKR RNA binding sites by dsRNA sequestration, direct obstruction of these sites (e.g., Vaccinia virus, Influenza virus), or interference with the phosphorylation of eIF2α (e.g., Human Immunodeficiency Virus 1) (Dzananovic et al., 2018). Specifically, adenovirus and Epstein-Barr virus transcribe dsRNAs with structural elements required for binding the dsRBMs and a stem-loop structure that inhibits PKR autophosphorylation (McKenna et al., 2006; Wahid et al., 2009; Dzananovic et al., 2014).

In addition to its ability to sense dsRNA, primarily of viral origin, PKR is also activated in response to endogenous RNA. Many of these are non-coding RNAs and/or regulatory RNAs such as microRNAs (miRNAs). For example, the non-coding nc886 miRNA functions as a suppressor of PKR by interacting with it directly (Lee et al., 2011), and its expression is increased in some malignancies but reduced others (Lee et al., 2016). In accordance, its suppression or epigenetic silencing result in induction of apoptosis and increased expression of oncogenes in certain models of cancer (Lee et al., 2014; Hu et al., 2017), and a protective effect in other models of cancer *in vitro* (Lee et al., 2016). Additionally, overexpression of miR-29b in developing cerebellar granular neurons confers protection against ethanol neurotoxicity leading to apoptosis through the SP1/RAX/PKR cascade (Qi et al., 2014). Another example is the long non-coding RNA HOX antisense intergenic RNA (HOTAIR), whose overexpression in keratinocytes resulted in increased expression of PKR and, as a result, decreased cell viability, increased levels of apoptosis, and increased expression of inflammatory factors in ultraviolet B (UVB)-treated cells (Liu and Zhang, 2018). Furthermore, a recent study has shown that PKR binds other non-coding RNAs such as retrotransposons, satellite RNAs, and mitochondrial RNAs (which can form intermolecular dsRNAs through bidirectional transcription of the mitochondrial genome). In fact, in a screen for molecules which bind PKR, done using the formaldehyde-mediated crosslinking and immunoprecipitation sequencing, mitochondrial RNA constituted the majority of endogenous molecules that bind PKR (Kim et al., 2018). In addition, PKR has been proposed to bind dsRNAs formed by inverted Alu repeats (IRAlus), upon disruption of the nuclear membrane in mitosis, leading to the phosphorylation of eIF2α in this phase of the cell cycle (Kim et al., 2014).

## PKR in the Brain

### Neurodegeneration

In the past two decades, increased levels of PKR phosphorylation have been detected in the brains of patients with HIV and neurodegenerative diseases such as Alzheimer’s disease (AD) (Chang et al., 2002), Parkinson’s disease, Huntington’s disease (Peel et al., 2001), dementia, and prion disease (Hugon et al., 2009). Furthermore, elevated levels of p-PKR and p-eIF2α have been observed in several mouse and monkey models of AD, including wild-type mice and cynomolgus monkeys injected with Aβ_1-42_ oligomers (i.c.v.), APPSwe/PS1DE9 mice, and ApoE4 mice (Lourenco et al., 2013; Segev et al., 2016). In both AD and Huntington’s disease, PKR has been implicated as mediating an ER stress-induced cell death (Peel and Bredesen, 2003; Bando et al., 2005), and it is possible that this is also the case regarding other neurological disorders where PKR levels are elevated. In the case of AD, increased staining of phosphorylated PKR (p-PKR) and phosphorylated eIF2α (p-eIF2α) have been observed mainly in degenerating hippocampal neurons, partially colocalized with hyperphosphorylated tau, a major hallmark of AD, and p-PKR levels are increased in cerebrospinal fluid from patients with AD and mild cognitive impairment (Mouton-Liger et al., 2012; Hugon et al., 2017), in positive correlation with cognitive decline in AD (Dumurgier et al., 2013). According to another study, increased levels of p-PKR, p-eIF2α, and p-mTOR were found in peripheral blood lymphocytes derived from AD patients compared to healthy subjects, in correlation with cognitive decline, further supporting the use of these molecules as biomarkers for the diagnosis of AD progression (Paccalin et al., 2006). Moreover, sporadic cases of AD constitute approximately 95% of AD cases, while the rest are familial ones. The sporadic cases are hypothesized to result from interaction between genetic and environmental factors, such as virus infections. Indeed, a study that analyzed human genes involved in the cell response to the herpes simplex virus type 1 (HSV-1) in AD samples compared to healthy subjects identified a SNP (rs2254958) located on the 5′UTR region of EIF2AK2, the gene encoding to PKR. This SNP, found within an exonic splicing enhancer, was found to be associated with AD, and homozygous carriers showed slightly earlier onset of AD (3.3 years), especially in the absence of the APOE4 allele (Bullido et al., 2008).

It has also been shown that in neuroblastoma cells overexpressing PKR, incubation with Aβ peptide resulted in increased phosphorylation levels of eIF2α, concomitant with an increase in the number of apoptotic cells (Chang et al., 2002). In a reciprocal experiment, incubation of PKR^-/-^ neuroblastoma cells with Aβ peptide resulted in reduced levels of p-eIF2α and apoptosis, and in accordance, primary culture cells derived from PKR KO mice were less sensitive to Aβ-induced toxicity (Chang et al., 2002). Finally, treatment with C16, the most widely used PKR inhibitor, in 12-month-old 5XFAD AD model mice rescued fear memory deficits almost fully, and restored LTP impairment in these mice. This was shown to occur without affecting Aβ_1-42_ levels in these mice. Similar cognitive rescue effects were induced by C16 in Aβ_1-42_–injected mice in the novel object recognition task and LTP impairment (Hwang et al., 2017).

The link between neurodegenerative diseases and oxidative stress has been a prevailing dogma in neurodegeneration research in the past three decades. Recent studies suggest a link between oxidative stress and PKR. Specifically, the anti-oxidant drug gastrodin (a phenolic glucoside), which suppresses BACE1 expression has been shown to enhance long term memory in the Tg2576 mouse model of AD in the Morris water maze paradigm of spatial learning. While induction of oxidative stress using H_2_O_2_ in neuroblastoma cells led to increased levels of pPKR, p-eIF2α, and BACE1, in accordance with the literature, treatment with either gastrodin or a peptide PKR inhibitor prevented the increased elevation in all three parameters, indicating that gastrodin exerts its neuroprotective effect by inhibition of the PKR/eIF2α pathway (Zhang et al., 2016). Another study has identified PKR as an inducer of apoptosis in response to oxidative stress. The authors showed that oxidative stress induced by nicotinamide adenine dinucleotide phosphate reduced oxidase (NADPH oxidase; NOX), an enzyme activated downstream of ER-stress, leads to the activation of PKR and amplification of its downstream target CCAAT/enhancer binding protein homologous protein (CHOP), resulting in apoptosis (Li et al., 2010).

### Learning and Memory

Protein kinase R has also been directly implicated in learning and memory. Cumulative evidence suggests that *de novo* global protein synthesis is a prerequisite for the consolidation of labile, short-term memory into more stable, long-term memory (Rosenblum et al., 1993; Klann and Richter, 2007; Gkogkas et al., 2010; Alberini and Chen, 2012; Gal-Ben-Ari et al., 2012). Since the rate-limiting step of most protein synthesis through mRNA translation is the initiation phase, it is plausible that global protein synthesis during memory consolidation involves the eIF2α pathway. Global protein synthesis is increased when phosphorylation levels of eIF2α are decreased. Indeed, enhancement of long term memory has been shown in both mice and rats, in cortical- and hippocampal-dependent learning paradigms, using genetic and pharmacological methods for decreasing eIF2α phosphorylation directly or indirectly, by reducing expression levels or activity levels of any of its four regulatory kinases, including PKR. For example, eIF2α^+/S51A^ mice (where Ser51 is replaced with alanine, preventing the phosphorylation of eIF2α) show enhanced performance in hippocampal-dependent spatial memory and contextual and auditory fear conditioning, and cortical-dependent conditioned taste aversion (CTA). The reciprocal experiment of stereotaxic administration of Sal003 (a derivative of salubrinal, which inhibits eIF2α dephosphorylation) into the rat hippocampus resulted in impaired contextual fear learning (Costa-Mattioli et al., 2007). However, these findings may be ascribed to PERK, rather than PKR, since similar memory enhancement has been observed by PERK genetic reduction (viral vectors and PERK KO mice) or pharmacological inhibition (using PERK inhibitor GSK2606414) (Trinh et al., 2012; Ounallah-Saad et al., 2014; Trinh et al., 2014; Sharma et al., 2018; Zimmermann et al., 2018), and also in a mouse model of AD (Ma et al., 2013; Yang et al., 2016). It is important to note that the main kinase to determine the basal phosphorylation state of eIF2α in the brain and primary culture is PERK (80%), while the other three eIF2α kinases including PKR determine the remaining 20% (Ounallah-Saad et al., 2014). Below we list findings supporting the beneficial effects of inhibiting or suppressing PKR specifically.

Similarly, in rats, pharmacological inhibition of PKR using C16 (aka PKRi) resulted in enhanced cortical-dependent novel taste learning (insular cortex-dependent positive, incidental learning) and CTA (negative, insular cortex dependent taste-malaise associative learning) when administered either i.p. or stereotaxically into the insular cortex prior to the taste stimulus. Similar results were obtained in mice using these paradigms. This effect of C16 on memory enhancement was shown to be PKR-specific, since it did not occur when PKR^-/-^ mice were administered C16 either in the novel taste learning or the CTA paradigm. However, administration of C16 did not affect phosphorylation levels of eIF2α either in the hippocampus or the cortex, in WT or PKR KO mice (Jiang et al., 2010; Stern et al., 2013). This point has been neglected in the literature thus far, and is in line with the fact that PERK is the major kinase to determine levels of eIF2α phosphorylation in the brain (Ounallah-Saad et al., 2014). We deem it important to explicitly state that, contrary to our simplistic view, C16 administration usually does not decrease p-eIF2α levels either *in vivo* or in cell culture [e.g., Hwang et al., 2017; 5XFAD mice treated with Aβ_1-42_ and PKRi (0.335 mg/kg, i.p.)]. In fact, to the best of our knowledge, in certain cases, p-eIF2α levels can be decreased by C16 only by pushing cells to extreme conditions involving massive cell death, such as prolonged incubation with toxic agents and/or high concentrations of C16 *in vivo* [e.g., striatal quinolinic acid administration combined with C16 (600 mg/Kg, i.p.) (Tronel et al., 2014)], *ex vivo* [e.g., bath treatment of brain slices; C16 (50 μM) for 2 h (Stern et al., 2013)], or in culture [e.g., PKRi 500 nM (C16) in cerebellar granular neurons from rats treated with amprolium (1.5 mM) for 24 h; amprolium is a thiamine competitor and depletes its intracellular levels (Wang et al., 2007); hippocampal neurons treated with Aβ oligomers for 3 h and PKRi (C16) 1 μM (Lourenco et al., 2013)]. C16 has an IC_50_ of 210 nM (Jammi et al., 2003).

A major current advancement in neuroscience is the ability to zoom in molecularly on specific cell/neuronal types. Zhu et al. (2011) have shown that PKR^-/-^ mice or WT mice treated with PKR inhibitor C16 have enhanced long-term memory and synaptic plasticity in inhibitory neurons, while synaptic plasticity in excitatory neurons is unaltered. Furthermore, the authors demonstrated that IFN-γ was increased in PKR^-/-^ mice. In addition, hippocampal-dependent memory enhancement, as measured in the contextual fear conditioning paradigm, was observed following administration of PKR inhibitor C16 and was abolished in IFN-γ^-/-^ mice. In accordance, treatment of mouse hippocampal slices with C16 led to sustained L-LTP in slices derived from WT mice, but not IFN-γ^-/-^ mice. The authors concluded that IFN-γ mediates disinhibition, which underlies the enhanced cognitive performance and synaptic plasticity when PKR is suppressed genetically or pharmacologically (Zhu et al., 2011). Importantly, the effect downstream of PKR is unclear, since levels of eIF2α phosphorylation in general or in the relevant GABAergic neurons were not measured. Further research using neuronal-specific manipulation is needed to better understand the possible differential role of PKR and/or the eIF2α pathway in different neuronal subtypes.

Other studies have also shown the direct involvement of IFN-γ in learning and memory and in synaptic plasticity. For example, the production of IFN-γ is altered in many conditions accompanied by cognitive deficits. A recent study has shown that hippocampal-dependent tasks such as spatial memory and recognition memory are enhanced in IFN-γ KO mice (while other functions, such as motor function or anxiety, for example, are unaltered). These IFN-γ KO mice were also shown to have increased DG neurogenesis, along with enlarged dendritic trees, characterized by longer dendrites in this brain subregion, as well as changes in cell volume and number, restricted to the dorsal part of the hippocampus (Monteiro et al., 2016).

## PKR in Neuroinflammatory Processes

As mentioned above, PKR is activated by pro-inflammatory cytokines (e.g., TNF-α, IL-1, and IFN-γ) (Khandelwal et al., 2011), and in turn, activates inflammation-related pathways, including the pro-apoptotic c-Jun N-terminal kinases (JNK) pathway (Bonnet et al., 2000; De Felice and Ferreira, 2014) and the pro-inflammatory NF-κB pathway (by direct interaction with IκB, an inhibitor of the NF-κB β subunit) (Bonnet et al., 2000). Activated PKR enhances IFN-α/β expression by IRF3 activation (Zhang and Samuel, 2008) and contributes to IFN-α/β mRNA integrity (Schulz et al., 2010). Activation of both IFN-α/β and NF-κB occurs downstream of toll-like receptor 3 (TLR3) activation in response to dsRNA. The signaling cascade, as demonstrated using poly I:C, involves (TLR3)-mediated activation of NF-κB and MAP kinase through the signaling components TLR3-TRAF6-TAK1-TAB2-PKR (Jiang et al., 2003). Depending on the cell type and insult activating PKR, it also induces the release of pro-inflammatory IL-1β, IL-18, and high mobility group box 1 (HMGB1) protein (Lu et al., 2012). However, in addition to its pro-inflammatory activity, PKR also activates anti-inflammatory IL-10 (Cheung et al., 2005; Chakrabarti et al., 2008) and reduces CD8 T cell proliferation in several models (Grolleau et al., 2000; Kadereit et al., 2000). In addition, PKR promotes apoptosis by interacting with the Fas-associated death domain protein (Couturier et al., 2010; von Roretz and Gallouzi, 2010) and upregulation of the proapoptotic factor Bax (Balachandran et al., 1998).

Indeed, neuroinflammation and activation of microglia are molecular hallmarks of AD, alongside neuronal loss, Aβ senile plaques (which are surrounded by reactive microglia and astrocytes), and neurofibrillary tangles of hyperphosphorylated tau protein (Duyckaerts et al., 2009). In addition, it has been shown in mice that inflammation, even if external to the brain, may lead to neuroinflammation and increased brain levels of Aβ (Kahn et al., 2012; Krstic et al., 2012), whereas treatment of brain-external inflammation may halt or even reverse the progression of this neuropathology. Increased brain levels of Aβ, in turn, may lead to exacerbation of inflammation, since Aβ peptide can activate microglia and lead to further release of pro-inflammatory cytokines, e.g., TNF-α or IL-1β (Kahn et al., 2012; Krstic et al., 2012; Carret-Rebillat et al., 2015). PKR contributes directly to neurotoxicity by activating pro-apoptotic caspase 3 and caspase 8, as shown in Aβ-treated cells and the APPSLPS1 knock-in mouse model of AD (Couturier et al., 2010).

A recent study has uncovered at least some of the molecular mechanisms underlying PKR-mediated neuroinflammation. In this study, the authors injected lipopolysaccharide (LPS; present in bacteria and used to induce inflammation) intraperitoneally to WT or PKR-KO mice, and measured inflammation-related parameters in the cortex and the hippocampus. These authors showed that many of the inflammation-related parameters were PKR-dependent, since these phenomena were not observed in PKR knockout mice, as opposed to WT mice, including LPS-induced increase in hippocampal neuroinflammation (measured by IBA1, a marker of microglia activation), cytokine release (TNF-α and IL-6), as well as BACE1, Aβ_42_, and phosphorylated STAT3 (BACE1 transcription regulator) protein expression levels (Carret-Rebillat et al., 2015).

In another study using PKR KO mice, 7-week-old mice were challenged with intracranial administration (into the left hemisphere) of the neurovirulent JHM strain of mouse hepatitis virus, JHMV, which induces encephalitis. In this model, too, the increase in brain levels of pro-inflammatory genes observed in WT mice was prevented in PKR KO mice (e.g., *Il-6*, *Ccl5*, and *Cxcl10*) (Kapil et al., 2014). However, no such PKR KO vs. WT mouse differences were observed in the respective proteins encoded by these genes, or IL-1β levels (Taga et al., 2017). By contrast, other inflammation-related genes and their respective proteins were matched in the impaired pro-inflammatory response in PKR KO mice compared to WT mice, for example, IL-10 and TIMP1. Notably, IFN-γ levels (gene and protein) were higher in PKR KO mice compared with WT ones (Kapil et al., 2014). It should be noted that both IL-1β and IL-6 are upregulated following neuroinflammation, and both cytokines promote disruption of the blood–brain barrier (BBB) and recruitment of lymphocytes (Hopkins and Rothwell, 1995; Erta et al., 2012).

These data suggest that pharmacological inhibition of PKR or its downregulation, e.g., by a virus, may also protect against neuroinflammation and its exacerbation. Indeed, injection of C16 (600 μg/kg, i.p.), the currently most potent PKR inhibitor (IC_50_ = 210 nM; Jammi et al., 2003), to a rat model (10 weeks old) of acute inflammation, induced by unilateral stereotaxic administration of quinolinic acid (QA), decreased neuronal loss. Furthermore, it ameliorated neuroinflammation, as demonstrated by reduced levels of pro-inflammatory IL-1β and cleaved caspase 3, a marker of apoptosis and increased levels of anti-inflammatory IL-10 (Lu et al., 2012; Tronel et al., 2014). However, no significant differences were detected in TNF-α or IL-4 in the QA-treated animals following C16 treatment. In another study, treatment with C16 (100 μg/kg) was shown to prevent neonatal hypoxia-ischemia brain damages by inhibiting neuroinflammation, reducing pro-inflammatory TNF-α, IL-6, and IL-1β mRNA expression levels in neonate (7 days old) rats (Xiao et al., 2016). In both studies, less tissue damage was evident in C16-treated animals (Tronel et al., 2014; Xiao et al., 2016).

## The Role of PKR in Metabolism

### PKR in Whole-Body Metabolism

Evidence suggests that PKR constitutes the link binding metabolic stress, obesity, diabetes, and inflammation, although this is controversial across the literature. PKR is apparently involved in metabolism throughout the body, and increased phosphorylation of eIF2α is a hallmark of obesity and diabetes-related insulin resistance (Nakamura et al., 2010, 2014; Carvalho-Filho et al., 2012). Furthermore, in culture, PKR inhibits pancreatic β-cell proliferation (Song et al., 2015), whereas insulin treatment elevates PKR phosphorylation on tyrosine residues, while inhibiting poly I:C-induced PKR phosphorylation on threonine residues (Swetha and Ramaiah, 2015). Additionally, high glucose impairs insulin signaling by activation of the PKR pathway (Udumula et al., 2017), whereas PKR activation induces insulin resistance in peripheral tissues (Nakamura et al., 2010, 2014; Carvalho-Filho et al., 2012; Carvalho et al., 2013). In a recent study, PKR was shown to interact with TAR RNA-binding protein (TRBP) under conditions of metabolic stress, and that phosphorylation of TRBP results in the activation of PKR, which in turn leads to JNK activation. While overexpression of TRBP in obese mice resulted in exacerbation of glucose metabolism, inhibition of TRBP phosphorylation in the liver had beneficial effects, including improved insulin resistance and glucose metabolism as well as reduced inflammation (Nakamura et al., 2015).

In another study, where PKR KO mice were fed on a high fat diet (HFD), insulin levels were markedly higher compared to PKR KO mice fed on control diet or WT mice fed on either diet. However, no significant differences between WT and PKR KO mice fed on HFD were noted in other parameters measured, such as body weight or glucose levels (Taga et al., 2018). Similar findings were reported by Lancaster et al. (2016) regarding these parameters in HFD-fed PKR KO mice. However, Lancaster and colleagues reported that PKR does have a role in T-lymphocyte recruitment, and PKR KO mice had less T cells in adipose tissue, which was thought to protect them from inflammation. However, this was not the case, and the authors showed that genetic deletion of PKR did not protect these mice against saturated fatty acid-induced inflammation or inflammasome activation. Furthermore, contrary to the studies presented above, injection of poly I:C in order to increase PKR did not result in impaired glucose tolerance (Lancaster et al., 2016).

These contradictory findings may be explained by different transgenic mouse models used. The widely used PKR KO mouse models have a deletion either in the N terminal or C terminal of PKR, and cells derived from these models were shown to express truncated forms of PKR (Baltzis et al., 2002), which retain partial functionality, and studies have shown that different domains of PKR are required for its different functions. Indeed, the catalytic domain is necessary for suppression of mRNA translation regulation and induction of inflammation in response to excessive consumption of nutrients and energy (Garcia-Ortega et al., 2017); the dsRNA binding domain is required for the activation of PKR by snoRNA under conditions of metabolic stress (Youssef et al., 2015); and the protein binding domain of PKR (but not its dsRNA binding domain) is required for other functions, e.g., as an adaptor protein. For example, a catalytically inactive PKR with intact protein binding was shown to promote β-cell proliferation via the TRAF2/RIP1/NF-κB/c-Myc pathways (Gao et al., 2015). However, this finding is inconsistent with those reported by Song et al. (2015), where PKR was reported to inhibit β-cell proliferation through sumoylation-dependent stabilization of P53. Of note, most kinase inhibitor compounds, including inhibitors of PKR, target only the catalytic domain (Garcia-Ortega et al., 2017).

### PKR Metabolism in the Brain

Insulin plays a major role in orchestrating energy availability in the body, as well as in the brain, a high-energy demanding organ (Fernandez and Torres-Alemán, 2012). In recent years, it has become increasingly clear that metabolic dysregulation in the brain underlies cognitive disorders, including AD, now considered type III diabetes (de la Monte and Wands, 2008). Such metabolic dysregulation or metabolic stress may result from aging, particularly when combined with high caloric intake and lack of physical exercise, which may lead to health problems spanning obesity, cardiovascular diseases, and diabetes (see Figure 1).

Metabolic stress also plays a role in AD, inter alia, through the Apolipoprotein E (ApoE) protein, which plays a role in lipid metabolism and transport in the liver and the brain, including clearance of Aβ peptide from the synapse (Li et al., 1988). The ApoE4 ε4 allele (ApoE4) is currently the best studied risk factor for late-onset, sporadic AD, with a prevalence of 20% in the general population, compared to 50% in AD patients, although estimates vary between different sources (Ward et al., 2012).

A recent study examined the interplay of PKR, metabolic stress, and ApoE4. Following prolonged metabolic stress, induced via HFD (60% fat for 3 months), higher levels of anxiety behavior were observed in ApoE4 mice compared to control ApoE3 mice fed on the same HFD. Furthermore, maintenance on HFD led to poorer levels of metabolic parameters in ApoE4 compared to ApoE3 mice, resembling diabetes mellitus-like characteristics, manifested as more rapid weight gain, lower serum and plasma insulin levels, and higher serum glucose levels in ApoE4 compared to ApoE3 mice. Furthermore, this HFD protocol led to higher hippocampal levels of β-site amyloid precursor protein-cleaving enzyme1(BACE1) and p-eIF2α protein expression levels, as well as higher hippocampal levels of ATF4 mRNA in ApoE4 compared to ApoE3 mice (Segev et al., 2016). However, the increase observed in p-eIF2α protein expression levels may be ascribed to eIF2α regulatory kinases other than PKR, especially PERK, the predominant kinase to affect p-eIF2α, and the main kinase to respond to ER stress (Ounallah-Saad et al., 2014).

In another study, ApoE4 mice were shown to have poorer long-term memory compared to ApoE3 mice, as measured by freezing in the fear conditioning paradigm. However, a single-dose treatment with the PKR inhibitor C16 (0.335 μg/g body weight, 1 h before conditioning) resulted in restoration of long term memory in ApoE4 mice, with freezing levels similar to ApoE3 mice in the fear conditioning paradigm. In addition, hippocampal ATF4 mRNA levels were found to be higher in ApoE4 mice compared to ApoE3 mice, whose ATF4 levels were similar to those of C57BL/6 mice. Hippocampal ATF4 mRNA levels were further elevated in aged ApoE3 and ApoE4 mice (12 months old) compared to their younger (4 months old) counterparts. Similar findings were observed in humans, where ATF4 mRNA levels were higher in ApoE4 carriers (67–98 years old) compared to non-carrier age-matched controls (Segev et al., 2015).

While immune system aspects are discussed in the section above, the interplay of PKR, the immune system, and metabolism has been shown in several studies. For example, Aβ oligomers have been shown to remove insulin receptors from the neuronal surface, which in turn leads to activation of c-Jun N-terminal kinase (JNK). This is followed by inhibition of the insulin receptor substrate (IRS-1) and, in cultured hippocampal neurons, this inhibition was shown to be mediated both by JNK/TNFα and PKR (Bomfim et al., 2012). This is supported by the finding that elevated levels of serine phosphorylation of IRS-1 and activated JNK were found in brains of both AD and diabetes patients (Bomfim et al., 2012). In addition, JNK/TNFα signaling leads to peripheral insulin resistance (Gregor and Hotamisligil, 2011), and this may also be the case in AD. Recent studies have shown that while i.c.v. administration of Aβ_1-42_ oligomers to mice resulted in long term memory impairment, this impairment was prevented both in PKR^-/-^ mice and in TNFR^-/-^ mice, and mice treated with either PKR inhibitor C16 or TNF-α neutralizing antibody, infliximab (Lourenco et al., 2013; Hwang et al., 2017). Furthermore, treatment of hippocampal cultures with insulin prevented Aβ_1-42_ oligomer-induced phosphorylation of PKR (Lourenco et al., 2013).

## PKR in Endothelial Cells

Protein kinase R has multiple effects in the vascular system in general and in endothelium cells in particular. One mechanism through which PKR exerts its effect in the vascular system is by modulating the expression of adhesion molecules in endothelial cells in the vascular system, thereby leading to the onset and development of inflammation (Osborn, 1990; Carlos and Harlan, 1994). For example, the adhesion molecule E-selectin is expressed on endothelial cells during inflammation, and its transcription can be induced by TNF-α or IL-1 (Ghersa et al., 1992). The activation of E-selectin by these cytokines is mediated by NF-κB in conjunction with endothelial leukocyte adhesion molecule 1 (ELAM-1) (Schindler and Baichwal, 1994). In aortic endothelial cells derived from PKR^-/-^ mice, the induction of E-selectin by either TNF-α or PKR-specific inducer was attenuated, supporting the idea described above, that PKR functions downstream of TNF-α, and additionally, demonstrating that PKR mediates the role of the adhesion molecule E-selectin in inflammation. Furthermore, the authors showed that the attenuation of E-selectin activation in the PKR deficient mice was caused by a reduction in the formation of the NF-ELAM-1 complex, as well as reduced activation of NF-κB (Bandyopadhyay et al., 2000).

As mentioned above, PKR is activated in response to mechanical stress, and plays a central role in determining cell fate, whether toward apoptosis or survival (Gil and Esteban, 2000; García et al., 2007; Hugon et al., 2017; Watanabe et al., 2018). Furthermore, many of the factors known to promote or exacerbate congestive heart failure, which constitutes mechanical stress due to hemodynamic overload, are also known to activate PKR, including oxidative stress, Toll receptor activation, and low-grade chronic inflammation (Kadokami et al., 2001; Lu et al., 2010). In a recent study, it was shown that PKR activation is increased both in a model of chronic transverse aortic constriction in mice, a mechanically induced simulation of congestive heart failure, and in human samples of congestive heart failure. Moreover, PKR^-/-^ mice were protected from transverse aortic constriction-induced pulmonary congestion, cardiac dysfunction, elevation in inflammatory cytokines (TNF-α and IL-1β), and apoptosis (as measured by the TUNEL assay and mRNA and protein expression levels of pro-apoptotic Bax and Caspase-3) (Wang et al., 2014).

Many studies have shown that PKR plays a central role in angiogenesis, which in turn plays a central role in cancer, neurodegeneration, and inflammation, cardiovascular diseases, as well as age-related macular degeneration, a common cause for blindness in the elderly. In two independent studies using *in vitro* and *in vivo* models (each) for cardiovascular diseases mediated by hypoxia and mechanical stress caused by hemodynamic pressure, similar results were obtained, showing that PKR is necessary for angiogenesis and neovascularization. Specifically, Zhu et al. (2016) used the RF/6A rhesus choroid-retinal endothelial cell line, where hypoxia was chemically induced using cobalt chloride (CoCl_2_). In this system, PKR expression was upregulated in parallel with p-PI3K, p-Akt, and VEGF expression, all of which were downregulated using siRNA directed against PKR (Zhu et al., 2016). The authors demonstrated that PKR is upstream of p-PI3K, p-Akt, and VEGF using a p-PI3K inhibitor, which affected p-PI3K, p-Akt, and VEGF, but not PKR. In addition, the knockdown of PKR using siRNA in a co-culture of RF/6A and ARPE-19 cells resulted in decreased cell migration and tube formation, strongly implicating the necessity of PKR in the formation of vasculature. In a mouse model of choroidal neovascularization (CNV), which mimics age-related macular degeneration, PKR was colocalized with CD31, a marker of vascular epithelium. In this model, treatment with monoclonal antibodies directed against PKR resulted in decreased progression of CNV. These findings were supported by another study, focusing on peripheral artery disease (Zhu et al., 2015), where PKR^-/-^ mice were shown to have delayed blood flow recovery, with a 34% decrease in CD31 in the ischemic tissue, indicating a reduced number of endothelial cells. *In vitro*, the authors demonstrated in a model of human umbilical vein endothelial cells (HUVECs) that pPKR expression was increased in response to hypoxia, whereas inhibition of PKR using siRNA resulted in reduced microtubule formation and migration. Furthermore, VEGF expression was reduced both in PKR^-/-^ mice and in HUVECs treated with PKR siRNA, supporting the findings of the study above regarding the necessity of PKR for VEGF-mediated angiogenesis under hypoxia conditions.

Other studies have shown the role of PKR in angiogenesis in the context of hypoxia in tumors. For example, PKR was shown to function as a tumor suppressor, downregulating transcription of hypoxia-inducible factor 1α (HIF-1α) under hypoxia conditions. This was shown to occur by PKR-regulated activation of T-cell protein tyrosine phosphatase, which in turn suppresses signal transducer and activator of transcription 3 (Stat3) (Papadakis et al., 2010). The role of PKR in cancer is discussed in further detail below.

Aging, as a risk factor for cancer, cardiovascular diseases, and neurodegeneration, is also related to senescence of endothelial cells. A recent study has shown that PKR inhibition (either by siRNA for PKR or inhibition of its phosphorylation using 2-AP) can reverse palmitate-induced (an independent risk factor of cardiovascular diseases) senescence of HUVECs, by activating JNK. JNK activation results in inhibition of silent information regulator 1 (Sirt1), which serves as an anti-senescent factor (Li et al., 2018), by affecting downstream targets such as histones, transcription factors, and many other aging proteins, one of which is the tumor suppressor p53 (Volonte et al., 2015). Taken together, these studies point to PKR as an attractive target for the treatment of cardiovascular diseases.

## PKR in Cancer

### PKR, an Enzyme With Contentious Roles in Cancer

While the role of PKR in metabolic stress and brain function is well established and described above, the role of PKR in cancer biology remains a subject of debate, as both tumor-suppressive and tumor-stimulatory functions have been attributed to this enzyme. The attribution of different and even contradictory roles for PKR in tumorigenesis reflect its involvement in the regulation of diverse cellular processes which may differentially affect the cancer cell and its interaction with the tumor microenvironment. Such processes include cell autonomous events such as the negative regulation of protein synthesis through eIF2α phosphorylation or signal transduction through different pathways including NF-κB, which alter the susceptibility of the cell to apoptosis and modulate the expression of inflammatory cytokines. Thus, variations in PKR expression and activity are predicted to affect both cancer-cell-autonomous and non-cell-autonomous aspects of the developing tumor.

This duality of effects is predicted to be a source of differences in experimental results and in their ensuing interpretation, with dependence on tumor type, tumor stage, or experimental model. Thus, results may differ between *in vitro* vs. *in vivo* studies, immune-deficient vs. immunocompetent mouse models, and tumors driven or not by inflammation. Also, the regulatory role performed by PKR in transduction of oncogenic/tumor suppressor signals may serve as a source for dual roles in tumor progression. This is exemplified by the PKR-mediated activation of NFκB (Maran et al., 1994). PKR was shown to activate NF-κB in diverse cellular contexts, with a differential dependence on its enzymatic activity (Kumar et al., 1994; Bonnet et al., 2000; Bonnet et al., 2006). As NF-κB may have powerful, albeit contradictory (double-edged sword) roles in cancer, mediating either tumor promotion or tumor suppression in different tumor settings (Pikarsky and Ben-Neriah, 2006), one can imagine similarly dual roles for PKR. Moreover, the proposed non-enzymatic activity of PKR may support the distinction between pro- or anti-tumorigenic roles, alternatively resulting from modifications in PKR expression or activity. In this context, functional interactions between PKR and pro-tumorigenic signaling pathways [e.g., STAT3 (Shen et al., 2012) or v-mos (Dagon et al., 2001)] were proposed to inhibit PKR activity, resulting in a scenario where increased PKR expression may not necessarily coincide with its increased activity.

### Tumor Suppressor Roles of PKR

The notion that PKR functions as a tumor suppressor is supported by: (i) Cell growth inhibition upon PKR overexpression (Chong et al., 1992; Meurs et al., 1993). In this context, PKR-mediated regulation of cellular replication may occur either through the inhibitory effect of PKR on protein synthesis, an essential resource for cell growth, or through PKR-dependent phosphorylation of cell cycle regulators. Of note, the expression and activity of PKR are differentially regulated in the cell cycle (Zamanian-Daryoush et al., 1999), and exposure to dsRNA upon mitotic breakdown of the nuclear envelope and exposure of dsRNA was proposed as a mechanism for PKR activation in mitosis (Kim et al., 2014). However, both stimulatory (Kim et al., 2014) and inhibitory (Dagon et al., 2001; Yoon et al., 2010) roles have been proposed for PKR in mitotic progression, underscoring the putative dual role of PKR in cancer. (ii) PKR-mediated stimulation of apoptosis through different molecular mechanisms (Jagus et al., 1999; Gil and Esteban, 2000) including transcription- and translation-mediated increases in expression of receptors that mediate programmed cell death (e.g., Fas (CD95/Apo-1) and/or pro-apoptotic Bcl2 effector proteins (Balachandran et al., 1998), which result in increased caspase activity (Gil et al., 2002). (iii) Functional interactions between PKR and tumor suppressors which regulate apoptosis (e.g., p53). Indeed, PKR is a p53 target gene (Yoon et al., 2009). Moreover, Type I interferon increases expression of both p53 (Takaoka et al., 2003) and PKR, and PKR amplifies interferon β induction by dsRNA (McAllister et al., 2012). Furthermore, PKR and p53 physically interact, and PKR positively regulates p53 transcriptional activity (Cuddihy et al., 1999a,b), while p53 positively regulates gene induction by dsRNA (Hummer et al., 2001). Together, these data suggest that PKR and p53 are intertwined in a positive feedback loop. However, other studies show that dsRNA stimulates p53 degradation (Marques et al., 2005; Baltzis et al., 2007), suggesting a negative feedback loop involving p53 and PKR, and underscoring the complexity of their functional interactions. (iv) *In vivo* experiments demonstrating an inverse correlation between PKR expression and/or activity and tumorigenicity. For example, knockdown of PKR in HCT116 human colon cancer cells supported rapid tumor growth and resistance to genotoxic drugs in nude mice (Yoon et al., 2009). Similarly, expression of dominant-negative mutants of PKR resulted in malignant transformation of NIH 3T3 cells and endowed these cells with the ability to generate tumors in nude mice (Koromilas et al., 1992; Meurs et al., 1993; Barber et al., 1995). (v) Reduced expression and/or activity of PKR in tumors. For example, in head and neck carcinoma, PKR and the proliferation marker PCNA exhibited inversely correlated expression patterns, suggesting a proliferation-inhibitory role for PKR (Haines et al., 1998). Furthermore, in myelodysplastic syndrome (a slow growing form of blood cancer), deletion of chromosome 5q, and the ensuing lack of IRF1 expression, lead to reduced PKR expression (Beretta et al., 1996). In addition to reduced expression, inactivation of PKR, similarly to what occurs in cells of patients with B-cell chronic lymphocytic leukemia (Hii et al., 2004), was also suggested to support tumorigenesis.

### PKR and Tumor Promotion

The established roles of inflammation in cancer progression (Coussens and Werb, 2002; Hanahan and Weinberg, 2011), the pro-inflammatory nature of NF-κB signaling and its multiple roles in cancer development (Taniguchi and Karin, 2018), and the identification of PKR as a stimulator of NF-κB activity (Kumar et al., 1994; Maran et al., 1994; Bonnet et al., 2000, 2006) form a strong rationale for pro-tumorigenic signaling by PKR. Indeed, PKR has been identified as overexpressed and activated in several cancers including hematopoietic malignancies (Basu et al., 1997), breast cancer (Kim et al., 2000), melanoma, and colon cancer (Kim et al., 2002). For example, in melanoma, eIF2α phosphorylation and the ensuing translation reprogramming were recently described as drivers of phenotypic plasticity, invasiveness and therapeutic resistance in melanoma (Falletta et al., 2017). These studies suggest that eIF2α kinases, such as PKR, may switch melanoma from a proliferative to an invasive cancer cell, driving metastasis in this manner. Indeed, interference with PKR reduced the growth and metastatic potential of murine melanoma (Delgado André and De Lucca, 2007; André et al., 2014). Moreover, and in accord with a correlation between PKR expression and tumor progression, primary melanomas revealed minimal PKR immunoreactivity, while melanoma lymph node metastases expressed high levels of PKR (Kim et al., 2002).

**FIGURE 4:**
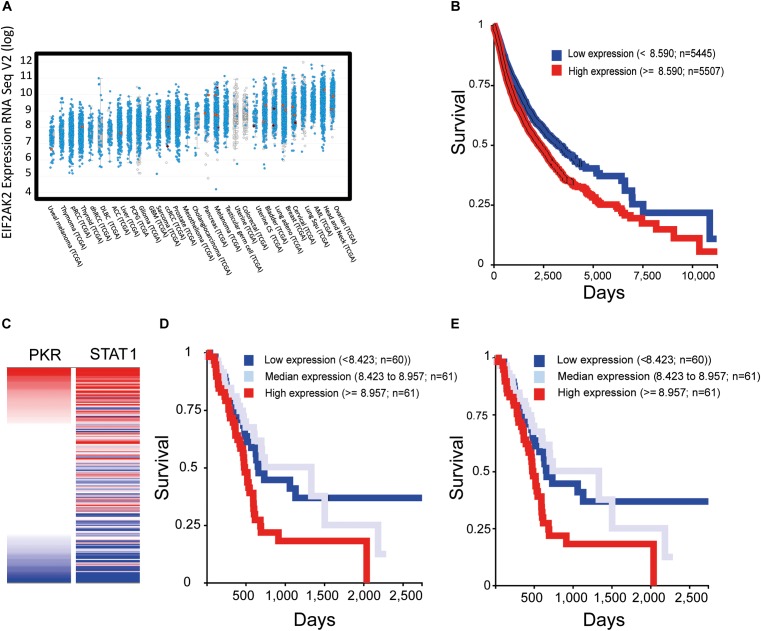
Increased expression of PKR correlates with activation of interferon-STAT1 signaling and with poor prognosis in multiple cancer types. To evaluate the expression of EIF2AK2 (PKR) in samples of cancer patients, we employed cBio Portal to assess studies of The Cancer Genome Atlas (TCGA). **(A)** Expression of EIF2AK2. Graph depicts the RPKM value of EIF2AK2 expression in different patient samples, ordered according to the median value of expression in the given cancer type. Blue puncta are samples where the EIF2AK2 sequence is wild type, red are samples in which EIF2AK2 is mutated. **(B)** Analysis of survival of cancer patients according to EIF2AK2 expression. Graph depicts the survival of patients (12830 patients form the PANCAN TGCA database, assessed and visualized with the UCSC Xena browser) classified according to a threshold of EIF2AK2 expression (blue, low expression; red, high expression). **(C)** Correlation of expression of EIF2AK2 and STAT1 in pancreatic cancer patients (196 cases, PAAD TGCA study, visualized with the UCSC Xena browser). **(D,E)** Analysis of survival of pancreatic cancer patients according to EIF2AK2 **(D)** or STAT1 **(E)** expression. Graph depicts the survival of patients (196 patients form the PAAD TGCA database, assessed and visualized with the UCSC Xena browser) classified according to threshold expression (blue, low expression; gray, median expression; red, high expression).

Recent transcriptomic studies in multiple cancer types (e.g., The Cancer Genome Atlas, TCGA) and their compilation into accessible public databases [e.g., cBio Portal, (Cerami et al., 2012; Gao et al., 2013)] allow for a global assessment of PKR expression in human tumors. The picture that emerges is one in which PKR (EIF2AK2) is broadly expressed across different cancer types, individual patients within a defined cancer type show considerably variable (up to 10-fold) levels of PKR expression (Figure 4A), PKR is rarely mutated, and 5–10% of patients show overexpression of PKR. Visualization of publically accessible TCGA data with the UCSC Xena browser^1^ shows survival data (Kaplan–Meier curves for overall survival) across multiple cancer types (TCGA PanCanAtlas, 12830 patients). These data revealed that higher levels of PKR expression correlated with poor survival (Figure 4B, *p* = 4.441E-16). For example, in pancreatic cancer (PAAD study, TCGA pancreatic cancer database, 196 cases), EIF2AK2 expression is considered as an unfavorable prognostic marker^2^; and data depiction with the UCSC Xena browser shows a negative correlation between PKR expression and survival (*p* = 0.001, Figure 4C). Indeed, expression of STAT1 (an interferon stimulated gene, and a mediator of interferon-transcriptional activity) and its correlation with survival in this cohort revealed a similar scenario to the one observed with PKR (Figures 4D,E). Together, these data support the notions of a pro-tumorigenic association of PKR expression and cancer, and of the regulation of its expression by JAK-STAT signaling in cancer cells. Of note, JAK-STAT signaling pathway is intimately associated with the transduction of signals from inflammatory cytokines (e.g., interferon gamma), suggesting that the pro-tumorigenic role of PKR occurs within the context of tumor-related inflammation.

PKR in cancer therapy. Due to its roles as a mediator of apoptosis and anti-viral responses, PKR expression and function have been implicated in two forms of anti-cancer therapy: chemotherapy and oncolytic virotherapy. In the former, PKR expression and activity have been positively associated with the therapeutic effects of 5-Fluorouracil [5-FU, (García et al., 2011)], doxorubicin (Peidis et al., 2011), bozepinib (Marchal et al., 2013), and histone deacetylase inhibitors [HDACi, (Peidis et al., 2010)]. Concerning oncolytic virotherapy, which aims at the specific infection and killing of cancer cells (oncolysis) and the activation of anti-tumor immunity, defects in interferon signaling in cancer cells expose these cells to viral oncolysis (Stojdl et al., 2000; Danziger et al., 2016). Specifically, defects in PKR activation were identified as a central mechanism by which oncogenic Ras enables oncolysis of transformed cells with oncolytic reovirus (Strong et al., 1998). In addition to oncolysis resulting from productive infection (a scenario which may benefit from defects in PKR expression or function), we have recently identified a novel form of viral oncolysis (oncolysis by non-productive viral infection, ONPVI) in which the combined exposure of interferon-responsive prostate cancer cells to a novel oncolytic virus (epizootic hemorrhagic disease virus-Tel Aviv University, EHDV-TAU) and interleukin-6, induced caspase-mediated cell death. ONPVI occurred in the context of STAT-1-dependent upregulation of multiple anti-viral gene products, including PKR (Danziger et al., 2018); opening the possibility that PKR may contribute to virally induced cancer cell death. Given the dependency of anti-immune checkpoint therapy on functional interferon-gamma/JAK-STAT signaling (Zaretsky et al., 2016; Sharma et al., 2017), and the positive feedback loop involving interferon signaling and PKR expression/function, we speculate that PKR may also play roles in this form of therapy. Together, these data suggest that the assessment of the status of PKR expression and function in cancer cells may be important for the choice of optimal therapeutic options, and that the development of means to manipulate its expression and function may have future applications in combination therapy settings.

## Tools for Inhibiting PKR

Taken together, the studies above point to PKR as a hub for co-morbidity and an attractive target for the treatment of metabolic diseases, cardiovascular diseases, neurodegenerative diseases, inflammation, and cancer. Moreover, when it comes to aging and correlated cognitive decline (Segev et al., 2015), PKR inhibition should serve both as an anti-neurodegenerative disorders agent and a pro-cognitive agent. The main obstacles to better understand PKR are (i) the available tools to inhibit PKR activity in general and specific functions of PKR in particular, (ii) the differences in expression levels between different cells, and (iii) the ability to manipulate PKR in specific cell types within a tissue. The most widely used pharmacological PKR inhibitor is the highly potent small molecule imidazolo-oxindole C16, also known as PKRi, which targets the ATP binding site of PKR. C16 has an IC_50_ of 210 nM *in vitro* (Jammi et al., 2003), and is typically used at doses of 210–500 nM *in vitro* for 1 h (Table 1). Incubation of cells with high concentrations of C16 induces high cell toxicity (see PKR in Learning and Memory section above). C16 has been successfully used by i.p. administration in mice and rats to elicit memory enhancement, indicating that the compound can cross the blood brain barrier. Table 1 shows that inhibition of PKR with C16 rarely inhibits eIF2α phosphorylation, the most known and cited substrate of PKR. It is clear that, in the brain, PERK is the dominant kinase to control basal levels of eIF2α phosphorylation (Ounallah-Saad et al., 2014); however, we do not know if this is the case in different neuronal subtypes (e.g., inhibitory versus excitatory neurons). Another less specific pharmacological inhibitor of PKR is the 2-aminopurine (2-AP) compound, which competes for ATP at the ATP binding site of PKR, and thereby inhibits its phosphorylation (Hu and Conway, 1993). This compound is less potent than C16, and is used *in vitro* at doses of 4–10 mM for 4 h (Endoh et al., 2009). Other inhibitors of PKR have been developed, although these were less potent than C16 (Weintraub et al., 2016).

**Table 1:** Summary of publications that used PKR inhibitor imidazolo-oxindole compound also known as C16 or Imoxim, which acts as an ATP-binding site directed inhibitor of PKR.

Field	Mode of administration	Concentration used	Model	Readout	Disease	Reference
Metabolism	Not given	5 μM for cells treatments	H9C2 cells treated with high concentrated glucose	Levels of pJNK/JNK↓,PKR↓and Caspase 3↓, mRNA levels of NFκB↓, JNK↓ and caspase-3↓. Measurements of ROS↓, nitrite levels↓, LDH↓	Diabetes	Udumula et al., 2018
	NA	5 μM (Imoxim)	NRK-52E cells treated with high fructose	Levels of PKR↓, caspase 3↓, Measurements of ROS levels↓. Apoptosis↓, JNK↓	Hypertension	Kalra et al., 2018
	Subcutaneous injection (Imoxim)	0.5 mg/kg (Imoxim)	Lean and obese male mice and MEF with TNFα	Glucose homeostasis↑ TNFα↓ and IL-6↓ mRNA in WTA. In MEF pPKR/PKR↓, pJNK/JNK↓, pIRS1^S307^/IRS1↓	Obesity	Nakamura et al., 2014

Immunology	NA	C16 400nM	THP1 macrophages	mRNA levels of GADD34↓, IL-8↓, IL-β↓	Inflammation	Dabo et al., 2017
	i.p.	100–500 μg/kg	Male BALB/C mice (7–8 weeks old) treated with intratracheal administration of LPS	Levels of TNF-α↓,IL-1β↓, IL-6↓, pPKR/actin↓, pIKK/IKK↓, pIkBα/IκB↓, pNFκB/actin↓, caspase3↓. Apoptosis↓ assessed by TUNEL↓ and pPKR↓ and pNFκB↓ by immunohistochemistry analysis. Analysis of lung injury↓ by hematoxylin and eosin stain	Acute lung injury	Li et al., 2017
	i.p.	150 μg/kg	Male SD Rats treated with Freund’s adjuvant	Limb swelling↓. Protein and mRNA levels a of HMGB1↓ and PKR↓ in blood and synovium	Rheumatoid arthritis	Wang et al., 2015
	NA	500 nM	mDC and BMDM derived from cybb^+/+^, cybb^-/-^ mice, pkr^+/+^ and pkr^-/-^	IFNβ mRNA↓ and pPKR/PKR↓	Parasite infection (*Chlamydia trachomatis*)	Webster et al., 2016

Neuroscience	NA	500 nM	SH-SYSY and UM1242-G cells exposed to EtOH	Cell viability↑	Alcohol use	Duncan et al., 2016
	i.p.	600 μg/Kg	Male Wistar rats treated with QA	Levels of pPKR/PKR↓, peIF2α/eIF2α↓. Assessment of neurodegeneration by hematoxylin and eosin stain↓. Immunofluorescence of cleaved caspase-3↓	Neuroinflammation	Tronel et al., 2014
	NA	1 μM	Hippocampus-derived neuronal culture treated with AβO	Synapse loss↓ assessed by immunocytochemistry against synapsin and PSD95	Alzheimer’s disease and diabetes	Lourenco et al., 2013
	i.p.	0.5 μ/kg	APPswePS1dE9	Levels of pPKR^T451^/PKR↓, pNFκB^S536^/NFκB↓, BACE↓ and TNFα mRNA↓, IL-1β mRNA↓	Alzheimer’s disease	Couturier et al., 2012
	NA	210 nM	Primary murine mixed co-cultures treated with Aβ42	Levels of pPKR^T451^/Actin↓, pNFκB^S536^/NFκB↓, pIκB^32/36^/IκB↓, pro-caspase3/cleaved caspase3↓ and levels of TNFα↓, IL-1β↓, IL-6↓	Alzheimer’s disease	Couturier et al., 2011
	i.p.	0.27 mg/kg	Mice treated with 3-NP	Assessment of neurodegeneration↓ by Cresyl violet staining	Huntington’s disease	Chen et al., 2008
	NA	500 nM	Cerebellar granular neurons from rats treated with amprolium (thiamine depletion)	Levels of peIF2α/eIF2α↓ and cell viability↑	Vitamin B1 deficiency	Wang et al., 2007
	i.p.	0.335, 3.35, 33.5, or 167.5 μg/kg	Sprague-Dawley rats (7 days old, 1, 2, 4, 6, 9, and 12 months old)	Levels of pPKR^Thr446^/PKR↓, peIF2α/eIF2α↓, pmTOR/mTOR (-), p70S6K^Thr389^/p70S6K(-) and pPERK/PERK(-)	Neuroprotection	Ingrand et al., 2007
	i.p. and local microinjection	167.5 μ/kg, 50 μM for hippocampal slices and local injection	Male Wistar rats, PKR-KO and WT (129SvEv) Hippocampal slices	CTA↑, NT↑ Levels of peIF2α/eIF2α↓, pPKR^Thr451/PKR^↓	Memory	Stern et al., 2013
	i.p.	0.1 mg/kg	Ifn-γ^-/-^ and WT CF57BL/6 mice	Synchronized EEG and inhibition↓, FC (Auditory and Context)↑	Memory	Zhu et al., 2011


*The inhibitor C16 was acquired from Calbiochem (cat. # 527450) or from Sigma Aldrich (cat. # I9785). ↑ up-regulation. ↓ down-regulation. (-) no change. NOR, Novel Objet Recognition; FC, Fear conditioning; MWM, Morris Water Maze; mDC, human monocyte-derived dendritic cells; BMDM, murine bone marrow derived monocytes; CTA, Conditioned taste aversion; NT, Novel taste; WAT, white adipose tissue; MEF, Mice Embryonic Fibroblasts; QA, quinolinic acid; NA, not applicable; AβO, Aβ oligomers.*

The PKR can also be inhibited by monoclonal antibodies and using genetic tools such as siRNA or viral vectors harboring an shRNA sequence directed against PKR, and both approaches have been successfully used *in vitro* and *in vivo* (André et al., 2014; Zhu et al., 2015, 2016). However, a new direction with promising high specificity is the use of biological, custom-designed peptides, whose advantages include high potency, high specificity, relative lack of toxicity, predictable metabolism, and selective targeting of specific functions (Kaidanovich-Beilin and Eldar-Finkelman, 2006; Eldar-Finkelman and Eisenstein, 2009; Fosgerau and Hoffmann, 2015). Indeed, some peptide drugs have already been FDA approved (Kaspar and Reichert, 2013). Still, peptides suffer from disadvantages, which include instability, high susceptibility to degradation, susceptibility to hydrolysis and oxidation, tendency for aggregation, short half-life, limited bioavailability due to their low membrane permeability, and consequently, the inability to administer them orally (Fosgerau and Hoffmann, 2015). However, in recent years there have been technological developments allowing to overcome some of the drawbacks of peptides, such as conferring membrane permeability by fusion to the Tat peptide or insertion of peptides into liposomes, micelles, nano-emulsions, or polymer nanoparticles to confer membrane permeability (Kaidanovich-Beilin and Eldar-Finkelman, 2006). Nevertheless, this strategy is still under development.

## Summary and Future

As can be clearly understood from the review above, we, the authors, recognize the complexity of PKR-mediated signaling in different cells and/or body/organs at different developmental stages and cellular compartments (Figures 2, 3). The main points we conclude from the many excellent papers summarized above are:

(1) PKR level and post-translation modifications are excellent biomarkers for neurodegenerative diseases (e.g., AD, dementia, Parkinson’s disease, Huntington’s disease, and prion disease) and cancer (Figure 4, based on open source data).

(2) Inhibition of PKR is predicted to be highly beneficial in age-related neurodegenerative diseases. PKR is positioned in the center of metabolic syndrome disease, including glucose or Aβ load and inflammation and its inactivation reduces the insult (Figure 1).

(3) PKR inhibition contributes positively and directly to cognitive function in young and old mice.

(4) Inhibition of PKR is beneficial in certain cases of cancer. However, here, the situation is more complex as the role of PKR in tumors (pro- or anti-tumorigenic) may differ according to tumor type and/or stage.

(5) PKR inhibition or deletion is not essential for an organism response to viral infection as detected in PKR KO mice or prolonged treatments with the best-known PKR inhibitor, C16, and thus has the potential to serve as medical treatment.

(6) Treatment with C16 following different stimulations in most cases does not affect eIF2α phosphorylation levels, although many publications are trying to explain the phenotypes of PKR inhibition via regulation of mRNA translation (Table 1). Moreover, brains of PKR KO mice do not show significant change in eIF2α phosphorylation. On the other hand, most papers do show a clear effect of PKR inhibition on the NF-κB pathway (Table 1).

(7) The recent findings that PKR detects not only exogenous, viral dsRNA but also endogenous dsRNA, such as mitochondrial RNA, point to it as a new target for reducing oxidative stress and apoptosis in disease states and specifically in neurodegenerative diseases.

We hypothesize that better understanding of PKR equilibrium and function in different scenarios, in addition to its ‘traditional’ role in cellular viral response, can be extremely important in understanding basic related biological processes such as inflammation, metabolism, aging, cancer, and brain function in normal and pathological states. Moreover, we predict that potent, non-toxic, specific inhibition of PKR function/s will serve as treatment for different diseases in certain situations. The most plausible steps in order test our hypotheses are:

(1) Identify small molecule inhibitors for PKR. Weintraub and colleagues (2016) employed a computational chemistry screening approach, which yielded interesting but unsatisfactory results. Screening small molecule libraries is the next reasonable step.

(2) Better understanding of the interplay of levels of PKR expression, function, and cell states.

(3) Identifying new tools (i.e., non-small molecule inhibitor), such as peptides, to inhibit specific functions of PKR.

(4) Understanding the role of PKR in specific cellular and subcellular compartments (e.g., neuronal dendrites) and cellular-specific context using genetics and/or pharmacokinetic tools.

We believe that the steps proposed above together with the new tools of omics and precision biology will allow better fundamental understanding of PKR functions to be translated into treatment of currently incurable diseases.

## Author Contributions

All the authors contributed equally to this work.

## Conflict of Interest Statement

KR serves as Chief Scientific Officer at Protekt Therapeutics Ltd. The remaining authors declare that the research was conducted in the absence of any commercial or financial relationships that could be construed as a potential conflict of interest.

## Footnotes

1 https://xenabrowser.net

2 https://www.proteinatlas.org
